# The role of self-esteem between perceived social support and positive Psycap in middle school students based on network analysis and mediation model

**DOI:** 10.1038/s41598-025-19279-x

**Published:** 2025-10-10

**Authors:** Lina Chen, Yingxia Li, Xiaohui Lu, Qingqing Miao, Zeqing Zheng

**Affiliations:** 1 School of Psychology, Capital Normal University, Beijing, China; 2 Department of Education, Hengshui University, Hengshui, China; 3 The Third Hospital of Shijiazhuang, Shijiazhuang, China; 4 College of Foreign Languages and Literature, Northwest Normal University, Lanzhou, China; 5 School of International Chinese Language Education, Beijing Normal University, Beijing, China; 6 State Key Laboratory of Cognitive Neuroscience and Learning, Beijing Normal University, Beijing, China

**Keywords:** Positive psycap, Perceived social support, Self-esteem, Network analysis, Mediation model, Human behaviour, Patient education

## Abstract

Positive psycap refers to the individual’s psychological state and psychological quality, which plays a vital role in the individual growth and development. This paper uses network analysis and a mediation model to explore the relationship among middle school students’ perceived social support, self-esteem, and positive psycap from the perspective of internal and external network structures and mechanisms. In this study, 736 middle school students (350 males) from two middle schools in Hebei Province participated in questionnaire surveys. The results revealed a positive correlation among three variables, especially with a moderately positive correlation between self-esteem and positive psycap scores.​ Network visualization analysis identified key nodes and edges, such as PSSS9, SE10, PPQ5, and PPQ19, as well as strong connections between PSSS3-PSSS9, SE9-SE10, and PPQ5-PPQ19. Stability and accuracy analyses confirmed the reliability of the centrality indices, and the resampling results were highly consistent with the sample.​ Furthermore, the mediation analysis indicated that perceived social support positively predicts positive psycap, and it also positively predicts self-esteem. And self-esteem can positively predict positive psycap. Perceived social support has a significant indirect effect on positive psycap through the mediating role of self-esteem, accounting for 32.4% of the total effect. These findings provide important insights into the mechanisms underlying adolescents’ psychological health. By fostering their perceived social support and self-esteem, their positive psychological qualities can be promoted, which is crucial for their growth and development.

## Introduction

Positive psychological capital, often abbreviated as " Positive PsyCap,” refers to an individual’s positive psychological states and qualities and plays a crucial role in their growth and development, particularly regarding adolescents’ psychological health and adaptability^1^. Psycap encompasses four core dimensions: self-efficacy, hope, resilience, and optimism^1^. In recent years, research on positive psycap has been increasingly prevalent, especially at the middle school stage, where adolescents face identity exploration, increasing academic pressure, and significant changes in emotional and social relationships^2,3^. Similarly, psycap is linked to better psychological and physical health in adults^4–6^.

Existing research has consistently shown that positive psycap positively impacts middle school students’ academic and personal growth, and are different from factors such as mental health. Higher levels of psychological capital are associated with better academic achievement, as students with higher self-efficacy, hope, resilience, and optimism are more likely to set challenging goals, persist in the face of difficulties, and adopt effective learning strategies^7^. From the perspective of the four dimensions of positive psycap, adolescents with a strong sense of self-efficacy will show stronger academic persistence and low depression^8^. It is hoped that by cultivating adaptive problem-solving and stress management skills, adolescents can cope with future challenges, such as college planning or career exploration^9^. Resilient adolescents show better emotional regulation and can use social support networks to reduce the risk of depression and improve classroom participation^10^. Optimism affects adolescent coping styles by promoting help-seeking behaviors and healthy lifestyle choices^11^. Furthermore, positive psycap is related to better psychological health, lower levels of anxiety and depression, and higher life satisfaction and well-being among middle school students^9,12,13^. Importantly, positive psycap can protect middle school students from the challenges and stressors they frequently encounter, such as academic pressure, peer relationships, and the transition to adulthood. Students with higher levels of psychological capital tend to display greater resilience in the face of adversity and are more likely to cope with and overcome these challenges adaptively^13–16^.

Many studies have noted the interplay between perceived social support and positive psycap. Perceived social support refers to an individual’s subjective evaluation of the availability and quality of support from their social network, and it is consistently associated with the positive psycap of adolescent populations. Students who perceive more social support from family, friends, and significant others often exhibit higher levels of self-efficacy, hope, resilience, and optimism, which are the core components of positive psycap^17,18^. This positive relationship between perceived social support and psychological capital is significant during the middle school years, a critical period of personal and social development for adolescents. When students feel supported and valued by their social network, they are more likely to develop the necessary psychological resources to cope with the challenges and stressors associated with this developmental stage^7,9^.

Furthermore, research suggests that the combination of perceived social support and positive psycap profoundly impacts middle school students’ overall well-being and adaptability. Students who perceive strong social support and higher levels of self-efficacy, hope, resilience, and optimism tend to exhibit better academic performance, higher life satisfaction, lower levels of anxiety and depression, and more effective coping strategies^16^. Psychological capital not only directly affects an individual’s mental health, but also indirectly affects the quality of life through perceived social support^19^. Improving an individual’s perceived social support can significantly enhance their psychological capital, thereby improving their mental health^20^. By fostering a supportive school and community environment and cultivating students’ psychological capital, schools can better assist adolescents in navigating the challenges and transitions they face during this critical developmental period^21,22^.

Research has also found a close link between self-esteem and positive psycap. Self-esteem refers to an individual’s overall evaluation of their worth and competence, and it is a fundamental aspect of psychological health and adaptive functioning. Middle school students with higher levels of self-esteem tend to exhibit greater positive psycap, including higher levels of self-efficacy, hope, resilience, and optimism^22,23^. This association is especially pronounced during adolescence because of a critical period of identity formation and social-emotional development. Self-esteem plays an important mediating role between psychological capital and anxiety^24^. The mediation of self-esteem and positive psychological capital can also alleviate the depressive symptoms of college students^25^. Students with a positive and stable sense of self-worth are more likely to develop the psychological resources needed to navigate the challenges and stressors associated with this transitional stage of life^7,26^. Furthermore, the combination of high self-esteem and positive psycap has a profound impact on middle school students’ overall well-being and academic performance. Adolescents who exhibit both high self-esteem and substantial psychological capital, tend to experience greater life satisfaction, lower levels of anxiety and depression, and better academic achievement^9,27^.

From the perspective of social cognitive theory^28^this interconnectedness can be understood through the reciprocal dynamics of environmental influences, personal factors, and behavioral outcomes. Studies have found that adolescents who perceive higher levels of support from their family, peers, and other important figures in their lives tend to exhibit greater self-esteem^29^. Longitudinal studies have also revealed the dynamic nature of the relationship between perceived social support and self-esteem, highlighting the importance of maintaining supportive relationships and a positive self-evaluation during adolescence^30^. In summary, existing research has predicted the relationships between social support and positive psycap^17,18^, between social support and self-esteem^17,18^ and between self-esteem and positive psycap^22,23^, so self-esteem may play a mediating role between perceived social support and positive psychological capital. In other words, perceived social support can directly and positively predict positive psychological capital, and can also partially or completely predict positive psychological capital through self-esteem.

The present study aims to delve into the network of relationships among middle school students’ perceived social support, self-esteem, and positive psycap, and focus on the potential mediating role of self-esteem in the relationship between perceived social support and positive psycap. Network analysis can effectively visualize the complex relationships between variables^31^, considering the mutual influence of multiple variables, and identify key nodes, thus providing an important basis for formulating effective intervention strategies. By unveiling the internal and inter-variable relationships among perceived social support, self-esteem, and positive psycap, and exploring the mechanisms underlying positive psycap, this study can provide more targeted strategies for promoting psychological health and intervention for middle school students. Furthermore, this research will incorporate network analysis and structural equation modeling to offer new perspectives and methods for psychological research on the relationships among psychological factors.

## Methods

### Participants

Before the study, the participants received thorough training to ensure they understood the test requirements and instructions. The research adopted a cluster sampling method, involving middle school students from two schools in Hebei Province, China. After obtaining informed consent from the schools, teachers, and parents, the students were asked to complete the survey anonymously. Before filling out the questionnaire, students also gave written informed consent and confirmed it again. The personal privacy information of all participants was strictly protected.

760 students (aged 13–17) participated in the survey, and 736 participants (96.84%) provided valid data. Of the participants, 350 were male (47.55%) and 386 were female (52.45%). The distribution across grade levels was as follows: 160 students in the seventh grade (21.74%), 159 in the eighth grade (21.60%), 122 in the ninth grade (16.58%), 147 in the tenth grade (19.97%), and 148 in the eleventh grade (20.11%).

All procedures were approved by Hengshui University (November 2020). All methods were performed by the relevant guidelines and regulations and adhered to the Declaration of Helsinki for the protection of human research participants. All participants and their parents voluntarily provided written informed consent.

### Measurements

#### Perceived social support scale

The model of perceived social support was first proposed by Zimet et al. in this study^32^ and later adapted to the Chinese context as the Perceived Social Support Scale (PSSS), which results showed a good model fit with GFI = 0.947, AGFI = 0.918, CFI = 0.971, and RMSEA = 0.077^33^. In this study, the PSSS model was used, which measures individuals’ subjective beliefs about social support from relatives, friends, and others. The scale consists of 12 items, and participants respond to each item on a 7-point Likert scale ranging from “completely disagree” (1) to “completely agree” (7), with a total score range of 12–84. Higher scores indicate a better perception of social support. In this study, the Cronbach’s alpha coefficient for the PSSS was 0.91.

#### Self-esteem

Self-esteem was assessed using the Rosenberg Self-Esteem Scale (SE)^34^. The scale consists of 10 items, rated on a 4-point scale from “strongly disagree” (1) to “strongly agree” (4). The total score ranges from 10 to 40, with higher scores indicating higher levels of self-esteem. In this study, the Cronbach’s alpha coefficient was 0.86, indicating good internal reliability of the scale.

#### Positive Psycap questionnaire

This study uses the Positive Psycap Questionnaire (PPQ) compiled by Kuo Zhang et al.^35^. The scale consists of 26 items and can be divided into four dimensions: self-efficacy, psychological resilience, hope, and optimism. In the confirmatory factor analysis, the model demonstrated favorable fit statistics. Specifically, the χ²/df ratio was 1.50, the RMSEA was 0.049, the ECVI was 2.86, the NNFI was 0.930, the CFI was 0.940, and the IFI was 0.940. These values indicate that the fit indicators either met or exceeded the ideal benchmarks, signifying that the model has a good fitting effect. Items 8, 10, 12, 14, and 25 are reverse-scoring questions. A seven-point Likert scale was used, with 1 indicating “totally disagree” and 7 indicating “totally agree”. The higher the score, the higher the level of an individual’s psychological capital. In this study, the internal consistency coefficient of the scale was Cronbach’s α = 0.93, and the Cronbach’s α of each subscale was 0.90, 0.76, 0.85, and 0.87, indicating that the internal consistency of the scale was good.

### Statistical analysis

Since the participants were of different genders and grades, no other covariates were included in the analysis, considering the study’s purpose and the model’s stability. The data analysis in this study is divided into three main parts. Firstly, IBM’s SPSS 24.0 was used for data cleaning, exclusion, and descriptive statistics to understand the data’s basic characteristics. Unlike the correlation analysis of the total score of variables, network analysis can show the relationships between the items in each variable in more detail, identify the important positions of key items in the entire network, and help to deepen the understanding of the association mechanism among variables. Secondly, the qgraph package^31^ in R version 4.2.1 (https://www.r-project.org/, Core Team, 2022) was employed for network analysis. The glasso (graphical lasso) network approach was used to describe the interrelationships among variables, with edges representing estimates of partial correlations and node degrees serving as centrality measures. Regularization was applied in the network analysis to avoid edge estimates^36^. Node degree analysis was performed using the sum of weights connected to the nodes, as other centrality measures (betweenness and closeness) are often unreliable in smaller samples^37^. Robust central nodes (symptoms) directly influenced many different nodes in the network. Additionally, the accuracy and stability of the network were analyzed.

Based on the content of the literature, the impact of social support on positive psycap is understood, and self-esteem may play a mediating role. Finally, the structural equation model analysis was conducted using the SPSS plugin Process to further explore the relationships among the three variables. If the 95% confidence interval contains zero, there is no statistically significant mediating effect at the 5% significance level.

### Check for common method bias

Harman’s one-factor test needs to examine common method biases due to the self-report data from each student. Unrotated factor analysis showed that eight factors were generated and could explain 63.99% of the total variance. The first principal factor explained 28.28% of the variance, which is less than 40%, indicating no serious common method bias in this study.

## Results

### Descriptive statistics and correlations

Table 1 shows the means, standard deviations, and Pearson correlation coefficients for the three variables of social support, self-esteem, and positive psycap among the high school students in this study. Overall, while the correlations between the three variables are not very strong, significant relationships are observed among them. Specifically, perceived social support is significantly positively correlated with self-esteem (*r = 0.17*,* p < 0.01*) and positive psycap (*r = 0.22*,* p < 0.01*). Moreover, a moderate positive correlation exists between self-esteem and positive psycap (*r = 0.45*,* p < 0.01*).

**Table 1:** Descriptive statistics and correlations among variables.

Variables	M	SD	1	2	3
1. Perceived Social Support	58.47	15.20	1		
2. Self-esteem	29.62	5.12	0.17^**^	1	
3. Positive Psycap	121.69	26.65	0.22^**^	0.45^**^	1

Note: **p < 0.01.

### Network estimation

**Fig. 1:**
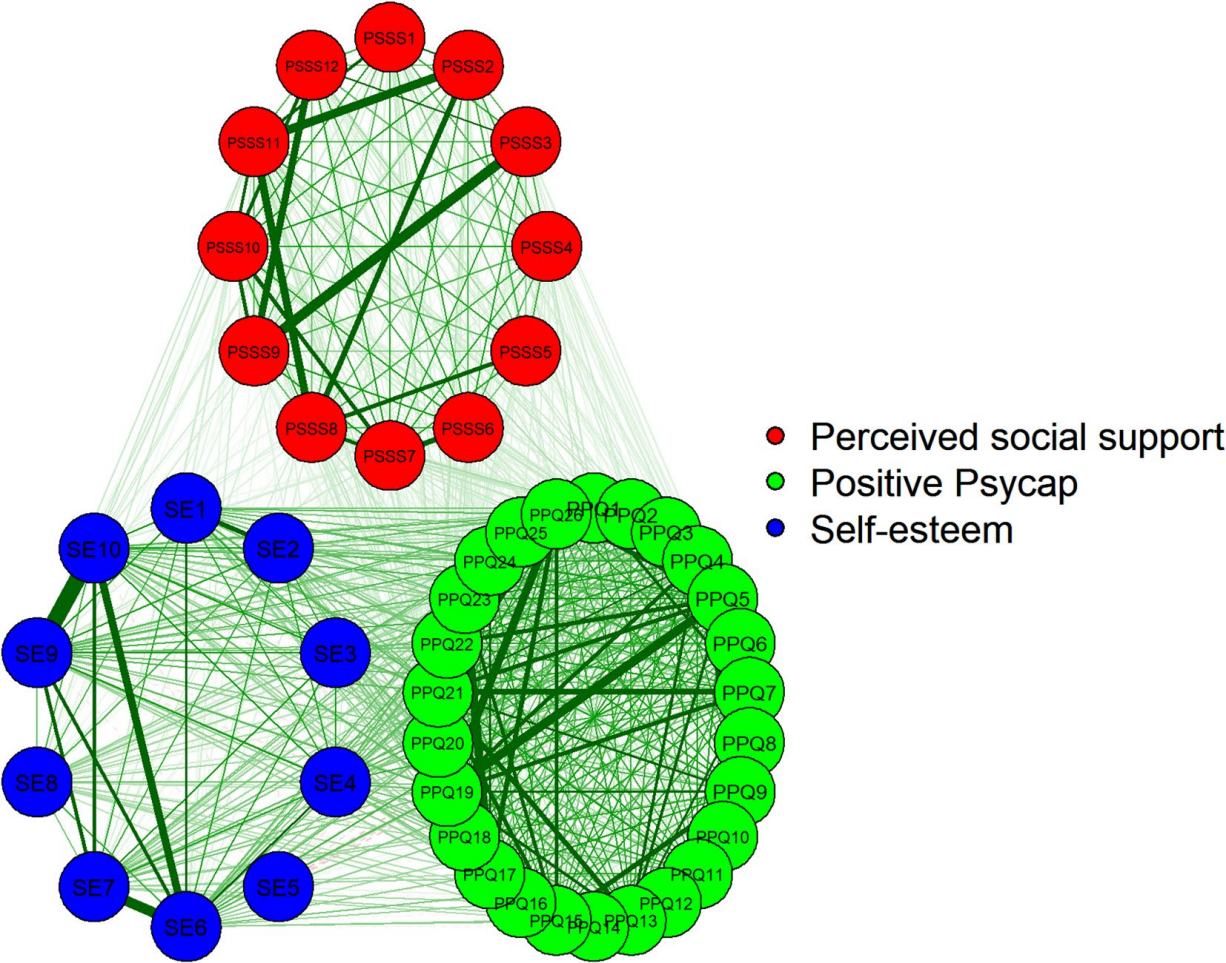
Network estimation structure and local network properties. Note: Each node represents an item, and each cluster of nodes with different colors represents the three variables. The red nodes represent perceived social support, the blue nodes represent self-esteem, and the green nodes represent positive psycap. The numbers on the nodes represent the items, and the lines’ thickness represents the strength of the associations.

In the study, the structure and centrality of perceived social support, self-esteem, and positive psycap are illustrated in Fig. 1. Through network visualization, we present the network structure graphically to enhance the understanding of the connections between nodes and the overall layout. This network consists of 48 nodes, with several nodes displaying strong centrality, such as PSSS9, SE10, PPQ5, and PPQ19. The eight strongest edges appear within the three communities. The three strongest edges in each community are as follows: PSSS3 and PSSS9 (*weight = 0.68*), SE9 and SE10 (*weight = 0.77*), PPQ5 and PPQ19 (*weight = 0.67*). Additionally, several non-zero edges between the three communities indicate interactions and associations among them.

**Fig. 2:**
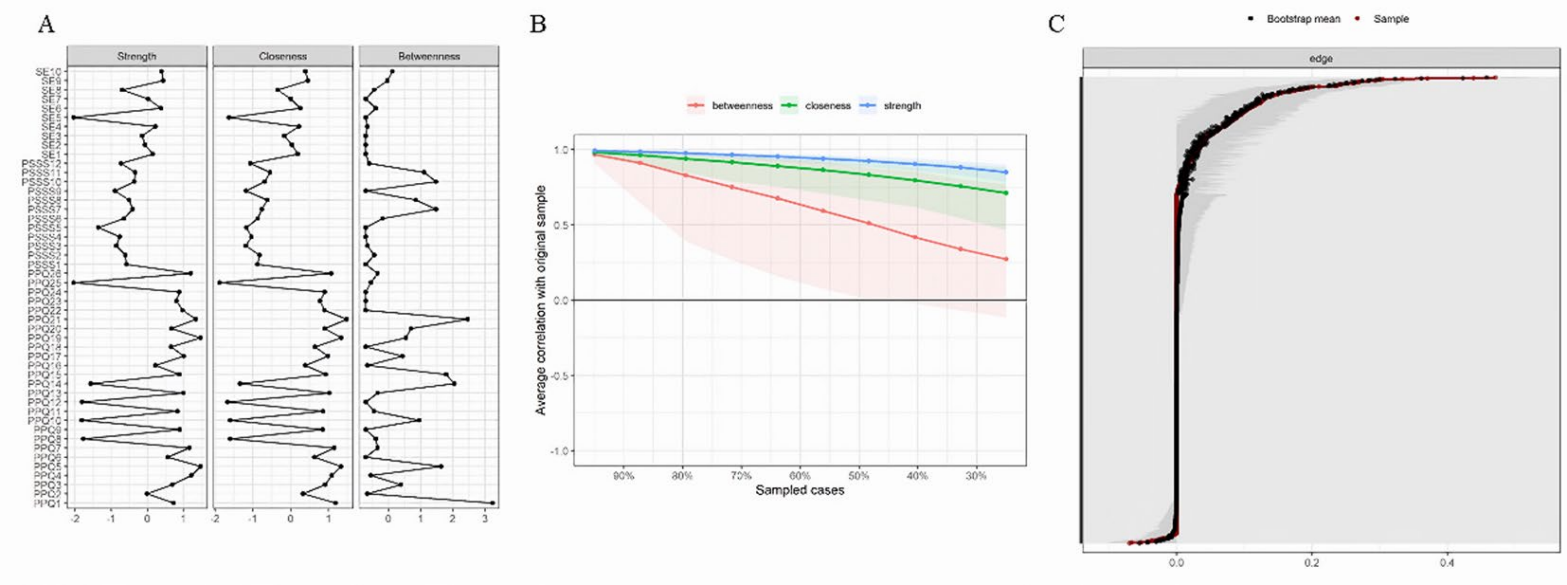
Network stability and accuracy. **A** shows the centrality indices of the network nodes, with the vertical axis corresponding to the items of the three variables, and the horizontal axis representing the standard Z-scores. **B** displays the stability analysis of the network centrality indices, with three colors corresponding to the three centrality measures, and the axes representing stability scores. **C** illustrates the accuracy of edge weight estimates using Bootstrap testing with a 95% confidence interval (*n = 736*). The red lines represent guided edge weights and indicate the values of the edges. The gray area represents the 95% confidence interval.

Figure 2A displays the centrality indices of the items for the three variables, helping us identify nodes that hold significant positions or play key roles in the network. Strength Centrality considers the strength of connections between nodes, with PPQ5 having the highest influence in this network. Closeness Centrality measures the average distance between nodes, and in this network, PPQ21 has a shorter distance to other nodes. Betweenness Centrality assesses the extent to which a node serves as a bridge for information transmission in the network. In this case, PPQ1 plays a crucial role in the information flow.

To evaluate the robustness and stability of the results, as well as the accuracy of the network, we used the R package “bootnet” (version 1.4.3)^38^. By performing bootstrap resampling, we generated different networks and calculated the stability of centrality indices in each network. As shown in Fig. 2B, all three centrality measures had correlations above 0.25 with the original sample under different sampling situations. The case-dropping bootstrap procedure shows that the Correlation Stability (CS) coefficients were 0.13 for betweenness centrality, 0.44 for closeness centrality, and 0.75 for both edge and strength centrality to retain a correlation of 0.70 in at least 95% of the samples. Figure 2C shows that the bootstrap confidence interval analysis was conducted to assess the stability of edges for network accuracy, and the results indicated a high level of overlap between the resampled means and the original sample.

Although the results above suggest the presence of important nodes and edges in this network structure, the stability and accuracy analysis of centrality indices demonstrates that this structure is reasonable. Further comprehensive analysis can be conducted to explore the relationships among the three variables.

### Testing for the mediation model

The above results show that the network analysis structure of the three variables is reasonable. Therefore, when further analyzing the three variables, considering the requirements of the process, the various items are integrated into explicit variables for analysis. Table 2 shows the mediation analysis results. The results showed that, in the first step, perceived social support positively predicted positive psycap, *β = 0.22*, *p* < 0.001 (Model 1 in Table 2). In the second step, perceived social support positively predicted self-esteem, *β = 0.17*, *p* < 0.001 (Model 2 in Table 2). In the third step, after analyzing the intermediary variable self-esteem, self-esteem can also positively predict positive psycap, *β = 0.42*, *p* < 0.01 (Model 3 in Table 2).

**Table 2:** Testing the mediation effect of self-esteem.

Regression model (*N* = 736)			*R*	*R* ^2^	F (df)	β	t
Model 1	PPQ		0.22	0.05	37.37*** (1)		
		PSSS				0.22	6.11***
Model 2	SE		0.17	0.03	21.38*** (1)		
		PSSS				0.17	4.63***
Model 3	PPQ		0.47	0.22	105.24*** (2)		
		PSSS				0.15	4.50***
		SE				0.42	12.84***

Note: ***p < 0.001.

**Fig. 3:**
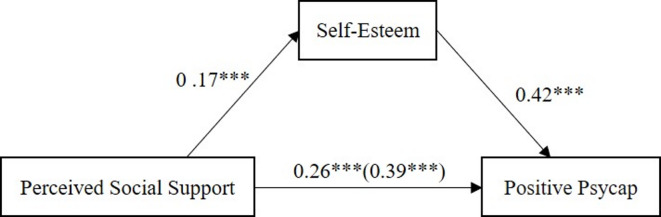
The mediation model.

Finally, the biased corrected percentile bootstrap method was used to explore the indirect effect of perceived social support on positive psycap through self-esteem, and it was found that the indirect effect was significant, with *ab = 0.13*,* SE = 0.03*,* 95% CI = [0.07*,* 0.18]*, as shown in Table 3. Therefore, self-esteem partially mediates the relationship between perceived social support and positive psycap, and the mediating effect accounts for 32.40% of the total effect. The diagram of the mediation model and its coefficients is shown in Fig. 3.

**Table 3:** The bootstrapping analysis of the mediating effects.

Types	Effect	BootSE	Boot LLCI	Boot ULCI	Proportion
Total effect	0.39	0.06	0.26	0.51	100.00%
Direct effect	0.26	0.06	0.15	0.37	67.60%
Indirect effect	0.13	0.03	0.07	0.18	32.40%

Note: Total effect: c = c’ + a*b, Direct effect: c’, Indirect effect: a*b.

## Discussion

The findings of this study underscore significant relationships among perceived social support, self-esteem, and positive psycap in adolescents, consistent with earlier research^12,34,39^. This suggests that adolescents who perceive higher levels of social support from their friends, family, and significant others also tend to exhibit higher levels of self-esteem and positive psycap. Notably, a significant positive correlation exists between psychological capital and social support among college students^40^. While the observed correlations are not exceedingly strong, they are meaningful and suggest avenues for further exploration. The internal dynamics within each variable and their interconnections remain complex, warranting additional analysis through network analysis and structural equation modeling. These approaches can provide deeper insights into how these constructs interact and influence each other.

This study utilized network analysis to explore the intricate relationships among perceived social support, self-esteem, and positive psycap in adolescents. The network analysis revealed that specific nodes, such as PSSS9, SE10, PPQ5, and PPQ19, exhibit strong centrality, indicating their pivotal roles within their respective communities. In the PSSS, students can rely on their friends when difficulties arise. In SE, students often feel worthless. In PPQ, they are confident in themselves and pursue their goals with assurance. Strong edges, particularly between nodes like PSSS3 and PSSS9, SE9 and SE10, and PPQ5 and PPQ19, underscore the robust connections within these groups. These findings suggest that specific elements of perceived social support, self-esteem, and positive psycap are tightly interlinked, potentially contributing significantly to adolescent development and well-being. This network analysis approach provides a more nuanced understanding that can help design targeted interventions to promote positive development in adolescents.

Identifying non-zero edges between different communities in the network analysis highlights the complex interaction and association between perceived social support, self-esteem, and positive psycap. This is consistent with findings from previous studies on children^10,41,42^. This intricate interplay suggests that enhancing one aspect, such as social support or self-esteem, could positively influence others, providing a holistic approach to improving adolescent mental health and adaptability. The strength centrality analysis identified PPQ5 as having the highest influence, corresponding to an item measuring self-efficacy, and indicating the important role of students’ self-confidence within this network of variables. Closeness centrality showed PPQ21 as occupying a central position in terms of connectivity, corresponding to an item measuring hope, and suggesting students’ energy affects other variables that influence other nodes in the network. Additionally, betweenness centrality highlighted PPQ1 as a key bridge for information flow, corresponding to an item measuring self-efficacy, and emphasizing its role in facilitating interactions across the network and the affirmation of oneself by others. These centrality measures suggest that the positive psycap construct plays a crucial role in the overall network, and those specific PPQ items may have a significant impact on the connectivity and dynamics within the network. This underscores the importance of considering the multidimensional nature of these psychosocial factors and their complex interrelationships when designing interventions to support adolescent well-being.

The network analysis confirms that the structure of these variables is reasonable, paving the way for further exploration through mediation analysis. The mediation analysis revealed that perceived social support significantly predicted self-esteem and positive psycap. Additionally, self-esteem significantly predicted positive psycap, and the indirect effect of perceived social support on positive psycap through self-esteem was significant, because social support may enhance adolescents’ ability to regulate negative emotions through modeling or explicit guidance from supportive figures, which in turn strengthens psycap^43^. This partial mediation suggests that social support directly influences psychological capital, which social cognitive theory emphasizes that environmental resources (like social support) can directly shape efficacy beliefs and goal-directed behavior^28^. However, self-esteem significantly enhances its impact, which aligns with existing literature emphasizing self-esteem’s critical role in bolstering resilience and well-being^34,44^especially in the mediating role of the relationship between psychological variables^8,45,46^.

To build on these findings, future research should explore additional mediators and moderators, such as coping strategies and emotional intelligence^47^to gain deeper insights into the mechanisms at play. Longitudinal studies are also recommended to observe how these relationships evolve^48^. Such research would provide valuable insights into the long-term impact of social support and self-esteem on positive psycap, helping to identify critical intervention periods. Self-esteem, as a core self-evaluation mechanism, reflects the overall evaluation and sense of value of the self. When individuals feel support and care from others, they will enhance their sense of self-worth and self-esteem, further affecting their emotions and cognition. Due to the limitations of this study’s sample and sampling process, we consider expanding the sample size and adopting more scientific sampling techniques in future studies to improve the stability and representativeness of the findings. Specifically, employing a multi-stage stratified sampling design that includes diverse geographical regions and varied school types would help capture the heterogeneity of adolescent experiences across different sociocultural contexts, strengthening the external validity of findings on the relationships among perceived social support, self-esteem, and psycap. And we combine longitudinal designs and multidimensional data to address the complexity of adolescent psychosocial development.

The findings of this study not only enrich and expand the existing theoretical model but also provide an essential basis for practical application. First, it clarifies the complex dynamic relationship between perceived social support, self-esteem, and positive psychological capital, offering a new perspective for understanding the internal mechanisms of adolescent mental health development. Self-esteem, as a key mediating factor, not only directly affects the cultivation of positive psycap but also enhances the role of social support in promoting psychological capital. This finding not only verifies existing theory but also expands upon it. In addition, these insights offer avenues for developing targeted interventions. By focusing on key nodes, like SE10 requires focused strategies to reframe negative self-perceptions using cognitive behavioral therapy, and mediation identified in the network, educators and mental health professionals can craft strategies that enhance positive psycap and self-esteem while effectively leveraging social support networks. In the future, we will include practical interventions aligned with contemporary developments based on the article’s results, particularly regarding the applications of AI-assisted mental health interventions that have gained significant traction in current practice.

## Data Availability

Data will be made available on reasonable request to Zeqing Zheng.
